# AFM Study of Nanoscale Membrane Perturbation Induced by Antimicrobial Lipopeptide C_14_ KYR

**DOI:** 10.3390/membranes11070495

**Published:** 2021-06-30

**Authors:** Sawinee Nasompag, Pawinee Siritongsuk, Saengrawee Thammawithan, Oranee Srichaiyapol, Panchika Prangkio, Terri A. Camesano, Chomdao Sinthuvanich, Rina Patramanon

**Affiliations:** 1 Interdisciplinary Graduate Program in Genetic Engineering, The Graduate School, Kasetsart University, Bangkok 10900, Thailand; sawinee.nas@gmail.com (S.N.); fscicds@nontri.ku.ac.th (C.S.); 2 Department of Biochemistry, Faculty of Science, Khon Kaen University, Khon Kaen 40002, Thailand; pawinee.siri@kkumail.com (P.S.); th_saengrawee@kkumail.com (S.T.); oranee_sr@kkumail.com (O.S.); 3 Department of Chemistry, Faculty of Science, Chiang Mai University, Chiang Mai 50200, Thailand; panchika.p@cmu.ac.th; 4 Department of Chemical Engineering, Worcester Polytechnic Institute, Worcester, MA 01609, USA; terric@wpi.edu; 5 Department of Biochemistry, Faculty of Science, Kasetsart University, Bangkok 10900, Thailand

**Keywords:** lipopeptide, rupture force, nanoscale, live bacterial cell, membrane

## Abstract

Lipopeptides have been extensively studied as potential antimicrobial agents. In this study, we focused on the C_14_-KYR lipopeptide, a modified version of the KYR tripeptide with myristic acid at the N-terminus. Here, membrane perturbation of live *E. coli* treated with the parent KYR and C_14_-KYR peptides was compared at the nanoscale level using AFM imaging. AFM analyses, including average cellular roughness and force spectroscopy, revealed the severe surface disruption mechanism of C_14_-KYR. A loss of surface roughness and changes in topographic features included membrane shrinkage, periplasmic membrane separation from the cell wall, and cytosolic leakage. Additional evidence from synchrotron radiation FTIR microspectroscopy (SR-FTIR) revealed a marked structural change in the membrane component after lipopeptide attack. The average roughness of the *E. coli* cell before and after treatment with C_14_-KYR was 129.2 ± 51.4 and 223.5 ± 14.1 nm, respectively. The average rupture force of the cell treated with C_14_-KYR was 0.16 nN, four times higher than that of the untreated cell. Our study demonstrates that the mechanistic effect of the lipopeptide against bacterial cells can be quantified through surface imaging and adhesion force using AFM.

## 1. Introduction

Natural lipopeptides (LiPs) are molecules consisting of a peptide linked to a lipid moiety commonly produced non-ribosomally in bacteria and fungi during cultivation in the presence of carbon sources. LiPs are considered among promising candidates for their potential use as antimicrobial agents and for vaccine therapy applications [1,2]. Compared to conventional antibiotics or antimicrobial peptides, bacterial resistance is generally rare in LiPs [1,2,3,4]. Therefore, synthetic LiPs have recently attracted considerable interest as the alternative to antibiotics. Most LiPs consist of a short linear or cyclic peptide covalently linked to a fatty acid (8–18 carbon chain) tail at the N-terminus. Among synthetic LiPs tested in the literature, the shortest LiPs with the highest bactericidal activity is composed of 2–4 amino acids linked to a fatty acid with a 8- to 16-carbon chain length [1,4,5]. The addition of fatty acid to a cationic peptide has been shown to promote a change in the secondary structure of the peptide [1]. The interaction between the amphiphilic peptide and the hydrophilic head of the fatty acyl chains of phospholipids leads to bacterial membrane insertion, and disruption of the physical integrity of the membrane, resulting in the leakage of cellular materials and eventually cell death [1,4].

The mechanisms of action of LiPs are similar to those of antimicrobial peptides (AMPs) where the bacterial membrane is a major target of LiPs. The general mechanism of AMPs involves the binding and permeabilization of the bacterial cell membranes. Most peptides are cationic and thus bind to anionic lipids in the target membranes via electrostatic interaction. Additionally, hydrophobic interactions contribute to the peptides’ affinity for the membranes where penetration through the lipid’s head groups unevenly distends and disrupts the membrane [6]. LiPs remain in oligomeric form in solution and bind to the cell surface via electrostatic interactions. At local concentrations above the lipid phase partitioning threshold, LiPs traverse through polysaccharide barriers via the microorganism’s self-promoted uptake mechanism to reach the outer and inner membranes. The partitioning of LiPs into cell membranes may kill pathogens via a membrane perturbation or a complete lysis of their membranes [1,6]. Although several studies have reported on the activity of LiPs, a few groups have demonstrated the mode of action of LiPs in detail and their selectivity for certain target membranes. Cell deformation and cytosolic leakage due to the antimicrobial effect are often shown via transmission or scanning electron microscopy studies, leaving behind the quantitative perspective of morphological changes [7,8]. Changes in cell topology and rigidity are an important key for visual evidence that can explain the antimicrobial mechanisms of LiPs [9,10,11].

Atomic force microscopy (AFM) has recently become a powerful technique for structural and morphological studies in microorganisms [9,12]. High-resolution topographical images of biological samples such as cells, microorganisms, and single molecules from AFM analysis provide significant information on structural, dimensional, and functional properties with superior detail with electron microscopy [8,9,13]. AFM allows the examination of samples in diverse environments without sample pretreatment, such as chemical fixation, dehydration, dryness, and/or metal coatings that may lead to changes in surface properties [8,14,15]. It can be used not only to image the surface topography at high resolution but also to locally measure the true three-dimensional data, adhesion force, and mechanical properties at the molecular level [8,9]. AFM imaging provides calibrated height information and allows accurate measurement of the surface roughness [9,16,17]. Importantly, a force–distance curve generated during the vertical movement of the AFM tip can explain a few mechanical and chemical properties of cell membranes such as membrane elasticity, turgor pressure, and hydrophobic and surface charge. The force–distance curves recorded at multiple locations of the plane provide maps of microbial physiochemical properties with nanoscale resolution. Additionally, adhesive maps and elasticity maps can be obtained from the data [12,16,18,19].

In recent years, progress has been made in understanding the surface properties of microorganisms using AFM. For example, Yang et al. studied the effect of silver ions on the bacterial membrane and successfully provided new insight into the antimicrobial mechanism of silver ions and the understanding of the relationship between the structure and function of the bacterial cell [8]. Formosa et al. demonstrated *Pseudomonas aeruginosa* as a model of a multidrug-resistant clinical strain for studying the differential effect at the nanoscale by two reference antibiotics. The organization at the nanoscale of the bacterial cell wall was examined using functionalized AFM tips to address changes in cell morphology, surface roughness, and elasticity after antibiotic treatment. The high resolution and sensitivity of AFM have brought novelties into the real-time effects of antibiotics and external agents in the cell envelope ultrastructure [20]. Fantner et al. developed special AFM cantilevers allowing high-speed tapping-mode AFM, and they observed the kinetics of antimicrobial peptide CM15 degrading individual *E. coli* cells at high resolution in real time at the nanometer level [21]. The same research group also successfully recorded high-resolution images with a force map of a nanoscale area. AFM-based approaches have also been used toward the morphological mapping of a wide variety of mechanical properties and the characterization of the functional response of biological systems under physiologically relevant conditions [17,22]. Recently, Beaussart et al. applied AFM-based single-molecule mapping with specific antibody probes to analyze the nanoscale distribution of the cell wall polysaccharides of the wild-type and the mutant strains of *Streptococcus agalactiae*. The AFM imaging and molecular mapping was represented as a platform for analyzing the molecular arrangement of the cell wall of the bacterial pathogens [18].

We previously modified KYR, a cationic tripeptide with myristic (C_14_) acid at the N-terminus. The resulting LiP, C_14_-KYR, exhibited a higher antibacterial property than the parent peptide, KYR [5], did. Here, we provided visual evidence for the antibacterial mechanism of C_14_-KYR against *E. coli* HB101 and for the morphological changes of the cell surface using AFM. The mechanistic comparison between the parent peptide, KYR, and LiP, C_14_-KYR, was elucidated. AFM images of *E. coli* HB101 showed that the C_14_-KYR induced dramatic changes on the cell surface, including shrinkage of the cell surface, separation of the cell wall and cytoplasm, and leakage of intracellular materials. In contrast, cells treated with the KYR revealed a slightly damaged surface, giving a similar pattern to the untreated cell. Additional evidence from SR-FTIR revealed marked structural changes in the membrane component after LiP attack. We clearly demonstrated in high-resolution images that the C_14_-KYR caused a higher degree of surface damage than its parent peptide.

## 2. Materials and Methods

### 2.1. Materials

Peptides were synthesized via Fmoc solid-phase peptide chemistry on rink amide-4-methylbenzhydrylamine hydrochloride salt resin by GL Biochem (Shanghai) Ltd. (Shanghai, China). The following peptides, the KYR and the C_14_-KYR, were dissolved in ultrapure water and further diluted to the desired concentration in either 0.9% (*w*/*v*) NaCl or phosphate-buffered saline (PBS), pH 7.4, depending on the experiments. Gram-negative *E. coli* HB101 used in antimicrobial studies was a gift from Camesano Laboratory, Worcester Polytechnic Institutes, Massachusetts, USA. All solutions were prepared with ultrapure Millipore water (Millipore Milli-Q plus, Billerica, MA, USA). The Luria-Bertani (LB) medium was purchased (Sigma-Aldrich, St. Louis, MO, USA) and sterilized under high-pressure steam at 121 °C for 30 min.

### 2.2. Sample Preparation for FTIR Analysis

The *E. coli* HB101 cell suspension (1 × 10^6^ CFU/mL) was centrifuged for 5 min. The cell pellets were washed with 0.9% (*w*/*v*) NaCl twice and re-suspended in 50 mL of 0.9% (*w*/*v*) NaCl. The cell was treated with 20 µM of C_14_-KYR (MIC) and 100 µM of C_14_-KYR (5x MIC) for 15 min at room temperature. Then, cells were washed three times with 0.1% (*w*/*v*) NaCl and resuspended in 0.1% (*w*/*v*) NaCl. The cell suspension was transferred onto Low-e Microscope Slides (MirrIR, Kevley Technologies, Chesterland, OH, USA) and was vacuum-dried for 30 min in a desiccator [23,24,25,26]. The cells on the slide were rinsed with distilled water and then vacuum-dried. This step was repeated to completely remove the salt. The washed and dried cell monolayer was stored in a desiccator until use.

### 2.3. Synchrotron Radiation FTIR Microspectroscopy (SR-FTIR)

The SR-FTIR experiments were conducted at the IR-end station of Synchrotron Light Research Institute (Public Organization) (SLRI, Nakhon Ratchasima, Thailand). The infrared spectra were collected using FTIR microspectroscopy coupled with an MCT detector cooled with liquid nitrogen over the measurement range from 4000 to 600 cm^−1^. Samples were analyzed in transflection mode, using the conventional internal IR source of a Bruker Vertex 70 spectrometer connected to the Bruker Hyperion 2000 microscope (Bruker Optics Inc., Ettlingen, Germany). IR signals were acquired from a 65 µm × 65 µm region, which covered more than 100 cells in the most homogeneous zone. The measurements were performed with a spectral resolution of 4 cm^−1^ with 64 scans co-added.

### 2.4. SR-FTIR Data Processing and Data Analysis

OPUS 6.5 software (Bruker) was employed to control the instrument, sample stage movements, spectral acquisition, and the post-acquisition processing of raw spectra. Integration was applied to the following intervals. Spectra from each group were analyzed using principal component analysis (PCA) to distinguish different chemical components of the samples using the Unscrambler 9.7 software (Camo Software AS, Oslo, Norway). In our experiment, the measurement produced 100 spectra of the untreated condition (normal cells), 137 spectra of the condition treated with a MIC of C_14_-KYR (MIC-treated cells), and 113 spectra of the condition treated with 5x MIC of C_14_-KYR (5xMIC-treated cells). The spectra were processed using the second derivative and were vector-normalized by the Savitzky–Golay method (3rd polynomial, nine smoothing points), and they were then normalized using extended multiplicative signal correction (EMSC) into one spectrum of each condition in the spectral regions from 1750 to 850 cm^−1^ [25,27]. Assigned bands in FTIR spectra represent the anti-symmetric and symmetric CH stretching from the lipids region (3000–2800 cm^−1^), amide I and amide II vibration of the protein region (1700–1500 cm^−1^), and functional groups (1300–900 cm^−1^) in nucleic acids (DNA and RNA), carbohydrates, phospholipids, proteins, and phosphorylated proteins.

### 2.5. Sample Preparation for Morphology Studies

The *E. coli* HB101 cell was prepared for imaging in each treatment condition as previously described with minor modification [25]. Briefly, a bacterial colony was picked and cultured in LB broth at 37 °C and 200 rpm overnight. The bacteria were further inoculated in LB broth and subsequently cultured at 37 °C and 200 rpm for 4–5 h until reaching a mid-logarithmic growth phase (OD600 ~ 0.6–0.7). The cells were harvested, washed, and resuspended in the 0.85% (*w*/*v*) NaCl. 10 mL of bacterial cell suspension (1 × 10^9^ cells/mL) was mixed with 300 µL of 100 mM of 1-ethyl-3-(3-dimethylaminopropyl) carbodiimide hydrochloride (EDC), pH 5.5 (Pierce), and it was then agitated for 10 min. After treatment with EDC, 300 µL of 40 mM of N-hydroxysulfosuccinimide (Sulfo-NHS), pH 7.5 (Pierce), was added to the bacterial suspension and mixed for an additional 10 min [28]. Prior to bacteria attachment, glass slides for AFM (MFP-3D-SA, Asylum Research, Santa Barbara, CA, USA) were rinsed with ultrapure water (18.2 MΩcm resistivity and <10 ppb total organic carbon), followed by sonication for 15 min. The glass slides were then immersed in 30% 3-aminopropyltrimethoxysilane in methanol (Sigma–Aldrich, St. Louis, MO, USA) for 20 min and rinsed with methanol and ultrapure water. To visualize the morphology of cells, bacteria cells (5–8 μL, 1 × 10^9^ cells/mL) were first exposed to either KYR or C_14_-KYR at 20 μM. The bacterial solution (5–8 μL, 1 × 10^9^ cells/mL) was added to each glass slide and agitated at 70 rpm for 1–2 h to promote bacterial lawn formation. To visualize the morphology of treated cells, 15–20 μL of either KYR or C_14_-KYR at 20 μM in PBS was added to the bacterial lawn. After incubation at room temperature, the cells were imaged by AFM.

### 2.6. AFM Imaging and Force Measurements

Imaging was performed with an MFP-3D Bio AFM (Asylum Research, Santa Barbara, CA, USA). The *E. coli* cell was imaged in solution using contact mode before and after treatment with KYR or C_14_-KYR. The microscope was equipped with a piezoscanner with a maximum scan range of 5 μm × 5 μm. Silicon nitride cantilevers (DNP-S, Veeco Instrument Inc., Santa Barbara, CA, USA) with a spring constant of ~0.12–0.16 N/m and a resonant frequency of ~5 kHz were used in imaging. The scanning speed was set to 0.5–1.0 Hz. Height, deflection, and phase images were obtained simultaneously in all experiments. Quantitative information from the AFM images were acquired via AFM analysis using the imaging software (Gwyddion). On average, 50 single force curves were acquired on top of the bacterial cell from at least 8 cells from two independent cultures for each condition. During force curve acquisition, the tip was made to gently touch the bacterial surface with a force of less than 5 nN to avoid breakage or deformation of the bacteria membrane. All retraction force curves were recorded at an interaction time of 1 s. The data analysis was performed using custom software written for MATLAB (The MathWorks, Natick, MA, USA) by using a procedure we previously described [29,30].

## 3. Results and Discussion

In order to provide visual and quantitative evidence for the mechanistic actions of the LiP, including pore formation on the bacterial cell surface and cell morphology at the nanoscale, the AFM technique was employed. In this study, we focused on the C_14_-KYR lipopeptide, a KYR peptide with amidation at the C-terminus and acylation with myristic acid at the N-terminus. Among the acylation of KYR with a 10- to 16-carbon chain length, C_14_-KYR exhibits antimicrobial activity toward both Gram-positive and Gram-negative bacteria with the highest selectivity index [5]. The *E. coli* HB101 was chosen as a bacterial model for studying changes in the bacterial surface after treatment with LiP. The characteristic change in surface cells after treatment with the antimicrobial agent was observed using an atomic force microscope. The insight event (at nanoscale) of the membrane disruption action on cells treated with the antimicrobial lipopeptide was obtained using imaging, characterizing the alteration in the biochemical properties involved with the antimicrobial lipopeptide.

### 3.1. Biochemical Alteration of E. coli Membrane Detected by the SR-FTIR Microspectroscopy

In order to understand the FTIR spectra for cell surface characteristics, the cell composition of bacteria is a prerequisite. Generally, Gram-negative bacteria have a thin peptidoglycan. The primary structure comprises parallel polysaccharide chains of N-acetyl glucosamine (NAG) and N-acetyl muramic acid (NAM) that are cross-linked by β (1→4) glycosidic bonds. The cross-bridge is most commonly composed of tetrapeptides such as L-alanine, D-glutamic acid, and meso-diaminopimelic acid (DPA). Gram-negative bacteria comprise the outer membrane (OM) of the peptidoglycan (PG), while the outside layer contains phospholipids, and the inner layer contains lipopolysaccharides (LPS). The outer layer consists of three basic regions: the O-specific side chain, the inner and outer core oligosaccharides, and a lipid anchor called lipid A [31,32]. When exposed to antimicrobial peptides, the bacterial cell surface is the first site of the attack following by membrane disruption, leading to cell death. Molecular damage at the membrane site results in changes in biochemical components of the membrane, i.e., loss of biomolecules, chemical bond breakage, or leakage of intracellular biomolecules such as DNA [33,34]. Basically, FTIR is a promising method that enables the analysis of the spatial distribution of biochemical components on the bacterial surface. However, analysis of FTIR spectroscopic data is complicated as absorption peaks often overlap with each other. Second-derivative spectroscopy is a technique that enhances the separation of overlapping peaks [33,35]. In this study, we employed synchrotron radiation FTIR (SR-FTIR) microspectroscopy to monitor the specificity of the second-derivative peaks for the change in cell surface components, and we followed a component alteration profile under any given condition.

**Figure 1:**
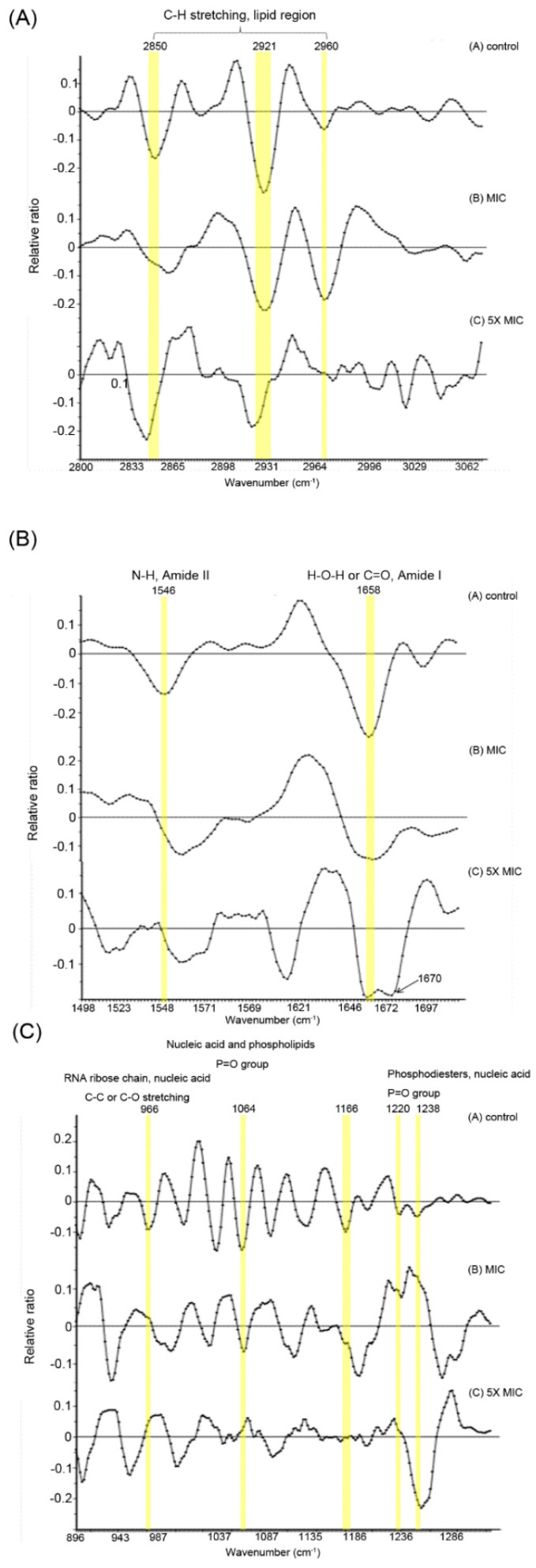
Second-derivative FTIR spectra of (**A**) untreated cells [n = 100], (**B**) cells treated with C_14_-KYR at MIC [n = 137], and (**C**) cells treated with C_14_-KYR at 5xMIC of C_14_-KYR [n = 113]. Assigned bands in FTIR spectra (highlighted in yellow) represent the lipid region (3000–2800 cm^−1^), protein region (1700–1500 cm^−1^), and carbohydrate and nucleic acids (1300–900 cm^−1^). Each second-derivative spectrum was an average from all spectra processed using the Savitzky–Golay algorithm and normalized with extended multiplicative signal correction (EMSC).

In our experiment, we attempted to discriminate the membrane component between untreated cells and C_14_-KYR-treated cells at MIC or 5xMIC using SR-FTIR microspectroscopy. Approximately at a given region of each condition, over 100 spectra were collected and analyzed. Figure 1 shows the representative second-derivative IR spectra in a certain range with assigned bands corresponding to the presence of macromolecules as follows: (A) lipid (3000–2800 cm^−1^), (B) protein (1700–1500 cm^−1^), and (C) carbohydrate and nucleic acid (1300–900 cm^−1^) [24,25,26,36]. When compared with the untreated cells, alterations in membrane components in all types of macromolecules were observed, as indicated by the differences in bands highlighted in yellow.

Figure 1A shows the spectra for the fatty acid and lipid region, including the *E. coli* profile and *E. coli* treated with C_14_-KYR. The obvious changes were related to shifting due to the deformation of C-H stretching. The treated *E. coli* at MIC C_14_-KYR was detected through modifications of bands at 2850, 2921, and 2960 cm^−1^, which were caused by C-H stretching from a hydrocarbon chain, i.e., fatty acid and lipid region. The main alteration in these regions of the MIC condition lightly shifted the peak, while 5xMIC C_14_-KYR addition was detectable with the decrease in the shift of the absorption peak, indicating the change in C-H stretching of the fatty-tail structure. The modification of the C-H vibration on the exposed bacterial profile is interpreted as bacterial lysis [26,34,36].

Figure 1B shows additional information for the component alteration profile under the treated condition derived from the main responsible bands for the protein region at 1700–1500 cm^−1^, which was dominated by the amide I and amide II of proteins and peptides [31,34,37]. Decreased peak intensities at 1546 and 1658 cm^−1^ (Figure 1B) were observed after treating *E. coli* with MIC and 5xMIC C_14_-KYR, corresponding to amide II and amide I, respectively. However, two bands remained in the untreated *E. coli* profile. These decreased peaks signify an increase in the deformed amide I due to the stretching vibration of C-O-C groups at the structure of proteins, as well as the responsible amide II region due to N-H bending with contribution from the C-N stretching vibrations of the peptide group. These results from *E. coli* exposed to C_14_-KYR conditions were introduced as dominated protein conformation alterations.

Furthermore, Figure 1C shows bands at 966, 1064, 1166, and 1220/1238 cm^−1^, corresponding to a mixture of functional groups in carbohydrates, lipids and phospholipids, nucleic acids, and phosphorylated proteins in the microbial membrane, respectively. All bands were observed in the untreated *E. coli* spectra. However, after being treated with MIC and 5xMIC C_14_-KYR, the intensity of the peak decreased. The deformation of the bacterial membrane can be the reason for the shift in the carbohydrate region, which shows P = O stretching in DNA, RNA, and phospholipids bands that shifted from and decreased the intensity peak at 1064 cm^−1^ in untreated *E. coli*.

The results showed that the spectral regions changed based on the degree concentration of alteration after the treatment of bacteria with C_14_-KYR, indicating that 5x C_14_-KYR treatment showed the most spectral modifications. The most remarkable differences were found in the protein and peptide regions. This could suggest that part of FTIR spectral changes were due to C_14_-KYR destabilizing the cell membrane by altering the structural conformation of membrane components, resulting in membrane disruption and leakage.

### 3.2. Morphology of E. coli HB101 after Treatment with KYR and C_14_-KYR Peptides

**Figure 2:**
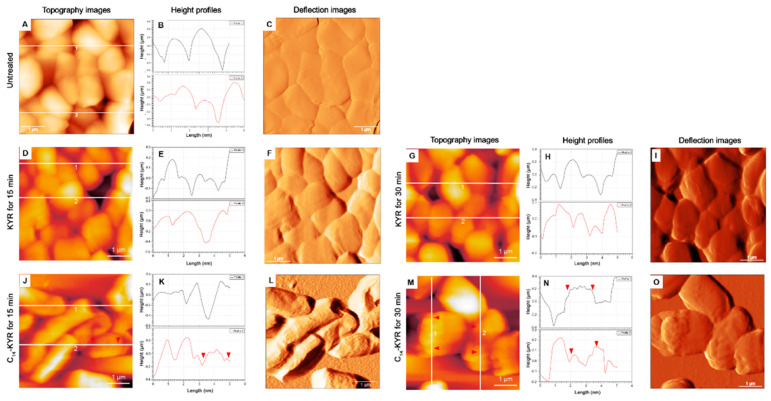
AFM images, height profiles, and deflection images of *E. coli* HB101 cell population in solution at mid-logarithmic phase before (**A**–**C**) and after treatment with either 20 μM of KYR (**D**–**I**) or 20 μM of C_14_-KYR (**J**–**O**) for 15 and 30 min, respectively. KYR slightly caused damage on the cell surface, while the rod shape was maintained after treatment for 15 (**D**–**F**) and 30 min (**G**–**I**). C_14_-KYR induced roughness on the cell surface after treatment for 15 (**J**–**L**) and 30 min (**M**–**O**). AFM images in 5 μm × 5 μm were performed using a silicon nitride cantilevers with a spring constant of ~0.12–0.14 N/m, a resonant frequency of 5 kHz, and a scanning speed 0.5 Hz.

Our previous study, C_14_-KYR, had the ability to disrupt the outer membrane of bacteria within 3 min with electrostatic bonding between the positively charged KYR and the negatively charged bacterial membrane. C_14_-KYR further induced inner (cytoplasmic) membrane permeability and also completely depolarized the bacterial membrane, indicating cell death within 10 min. Membrane depolarization was stably saturated after 15 min of treatment by lipopeptide. In contrast, the KYR peptide can bind to the outer bacterial membrane but cannot traverse across the membrane [5]. AFM images of the *E. coli* HB101 population in solution (see more detail in Section 2.5) were collected before and after exposure to 20 µM of each peptide for 15 and 30 min. Silicon nitride cantilevers were applied to the measurement morphology of *E. coli* HB101. The measurement data were acquired from the analysis of cells from ≥8 independent cultures. The properties of *E. coli* HB101 cells, including the size and roughness, are summarized in Table 1. As marked in the C_14_-KYR condition, the scan area of the surface cell shows an average roughness increased threefold after lipopeptide treatment, as well as increasing time of exposure. In contrast, the KYR peptide displayed an average roughness approximated to the untreated bacterial cell. Figure 2 illustrates changes in the cell surface induced either by KYR or C_14_-KYR at different time points. Topography images of *E. coli* cells revealed the height of cells and surface appearance. When the cell was not exposed to the peptides, its surface was relatively smooth without the appearance of ruptures or bulges, as shown in Figure 2A–C and Figure S2. The cell morphology appeared in a rod shape with some variations in cell width and length due to the different stages of bacterial growth. Upon the treatment of the KYR peptide, *E. coli* cells retained their rod-shaped morphology and their surface appeared smooth, as observed in Figure 2D–F even with prolonged incubation (30 min) (Figure 2G–I), indicating that the KYR peptide was unaffected by the alternate *E. coli* membrane. In contrast, treatment with C_14_-KYR for 15 min significantly increased the roughness of the cell surface, and cell shrinkage was observed, as indicated in Figure 2J–L. The cell morphology reduced in length for C_14_-KYR treatment following recorded data in Table 1. Due to C_14_-KYR affecting the cell membrane, this resulted in membrane disruption, cell shrinkage, and leakage. Deep groove-like lesions were clearly visible on the cell surface, as shown in Figure 2M–O (deep lesions are cell surface abnormalities that do not include smooth surface and surface roughness). Particularly in Figure 2N, the red marks on line 1 and 2 are the preferred roughness on the surface. These findings confirmed that C_14_-KYR induced more dramatic changes in cell surface roughness and morphology of *E. coli* HB101 than the KYR could. 

**Figure 3:**
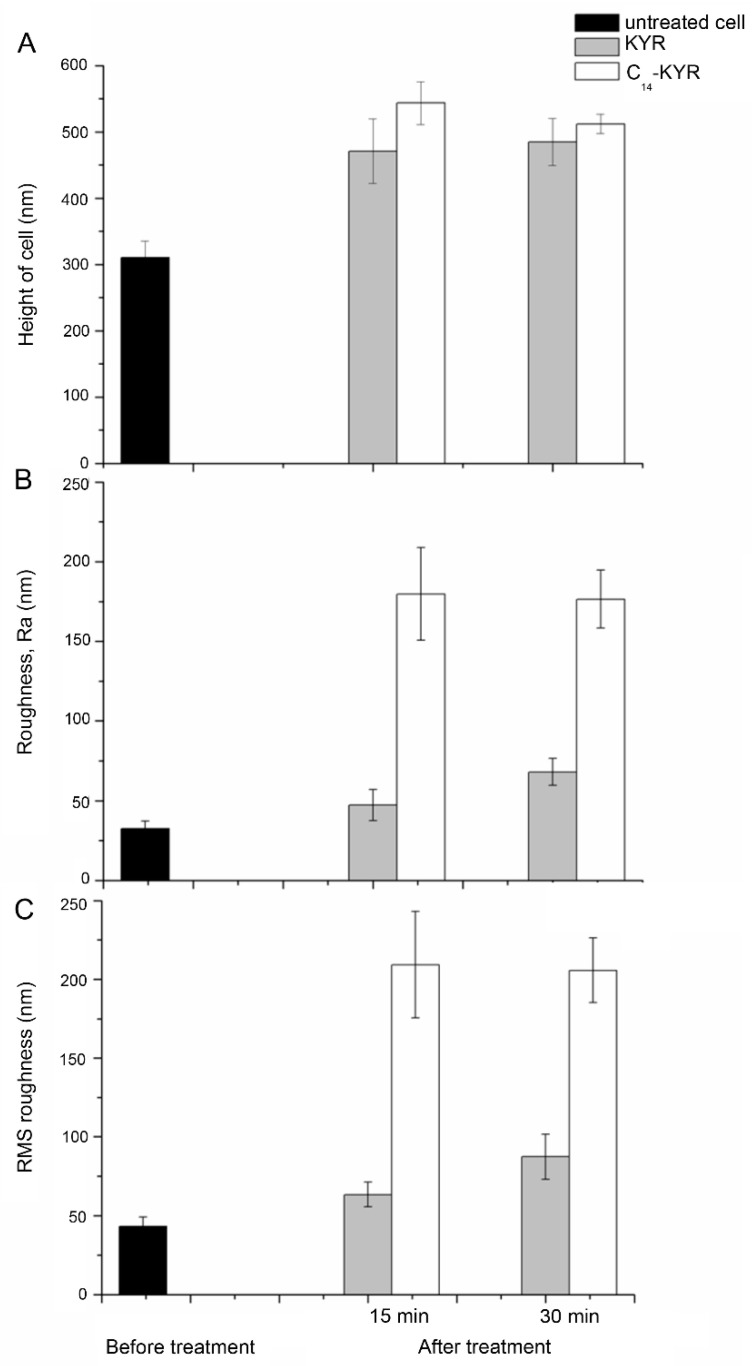
Surface roughness of *E. coli* cell in solution before and after treatment with either 20 μM of KYR or 20 μM of C_14_-KYR for 15 and 30 min. (**A**) Height of cell, (**B**) roughness (Ra), and (**C**) root-mean-square (RMS) roughness were measured from over 200 cells from more than 8 independent cultures for each condition.

To obtain further quantitative analyses on the cell surface roughness before and after the exposure to KYR and C_14_-KYR, we performed statistical analysis on the topography images at various length scales following Table 1. In both cases, the distribution of cell heights was well defined. The height (h) of the untreated cell was measurable at about 310.4 ± 25.2, but the average cell height increased significantly by approximately 160 and 230 nm after exposure to KYR and C_14_-KYR treatment for 15 min, respectively (as shown in Figure 3A and Table 1). The average roughness (Ra) and root-mean-square (RMS) roughness were analyzed using the power spectral density of the fast Fourier transform of the height images [34,35]. It is notable that the major differences in cell roughness were clearly observed when the cells were treated with the C_14_-KYR peptide, as compared to the untreated cells, as shown in Figure 3B,C. The Ra and RMS roughness of the C_14_-KYR-treated cell were substantially rougher (Ra and RMS of 179.67 ± 29.1 and 209.33 ± 33.8 nm, respectively) than the untreated cell. We noted that the increase in surface roughness correlated with the C_14_-KYR-induced change in morphology of the bacterial membrane, which eventually led to cell death. 

**Figure 4:**
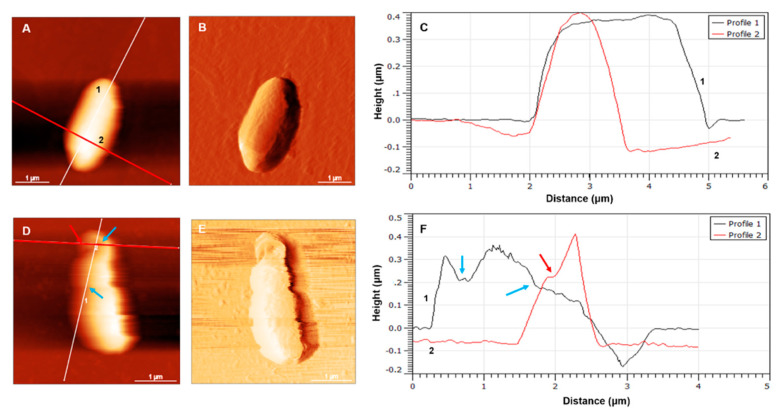
Topography of *E. coli* HB101 before (**A**–**C**) and after C_14_-KYR treatment (**D**–**F**). Upon the treatment of C_14_-KYR, the cell dimension became smaller due to cell shrinkage. Cell debris has accumulated at the border of the cell (**D**,**E**). Cross-sections of cells before and after treatment were observed, as well as variation in the cellular profile (right of **C**,**F**). The arrows in F indicate a major groove within the damaged cell. The cell height (h) was acquired from the analysis of a surface cell in the absence and presence of C_14_-KYR. Roughness (Ra) was measured from a 100 nm × 100 nm area on the cell surface from five different positions of each topography image. AFM images in 5 μm × 5 μm were performed using a silicon nitride cantilevers with a spring constant of ~0.12–0.14 N/m, a resonant frequency of 5 kHz, and a scanning speed 0.5 Hz.

**Figure 5:**
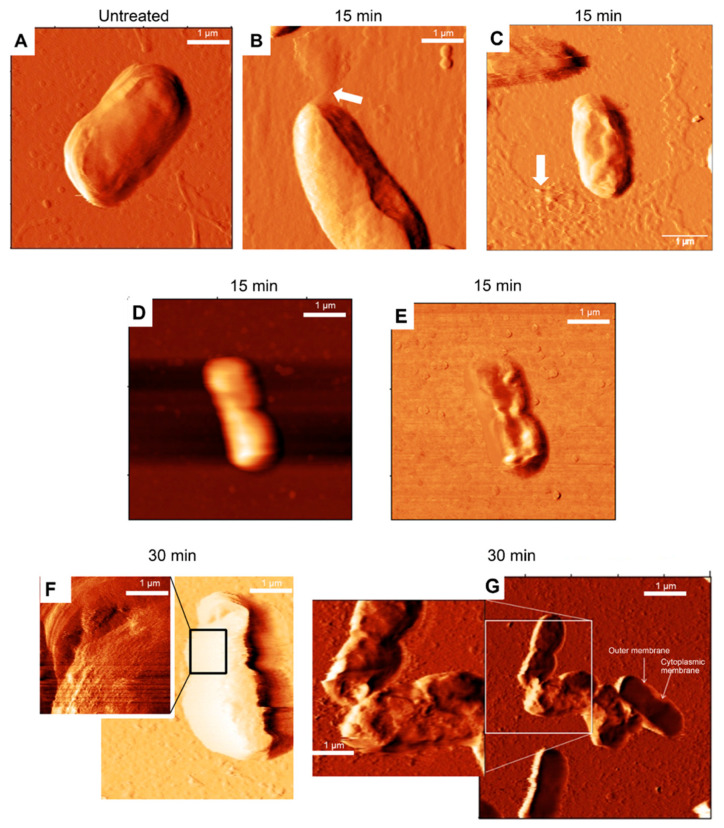
Effect of the C_14_-KYR on the bacterial morphology. AFM deflection images of *E. coli* cells incubated in the absence (**A**) and the presence of C_14_-KYR for 15 (**B**–**E**) and 30 min (**F**,**G**). After being treated with C_14_-KYR for 15 min (**B**,**C**), the intracellular fluid leaked out of the cell. The topography (**D**) and phase (**E**) images of the *E. coli* HB101 cell after 15 min of incubation with C_14_-KYR. The structure of the outer membrane of the C_14_-KYR-treated cell was maintained, but a change in the inner membrane due to cell shrinkage was observed in the phase image. The cell membrane became damaged and separated from the cell wall after 30 min of treatment (**F**,**G**). AFM images in 5 μm × 5 μm and 2.5 μm × 2.5 μm were performed using a silicon nitride cantilevers with a spring constant of ~0.13–0.16 N/m, a resonant frequency of 5 kHz, and a scanning speed 0.5–0.6 Hz.

The effects of the KYR and C_14_-KYR peptides on the single *E. coli* HB101 cell were visualized in three dimensions, as shown in topography images and height profiles (Figure 4). The height profiles of the cells provided quantitative measurements of the cell dimensions before and after C_14_-KYR treatment. After incubation with C_14_-KYR for 30 min, the morphology of the single *E. coli* HB101 cell dramatically changed, i.e., appearance of the groove surface, rough-like surface on the cell surface, and cell shrinkage. Figure 4D,E also illustrate cell debris, with organic waste leftover after a cell dies (lysis cell), accumulated at the border of the cell, which clearly appeared rougher than the untreated cell in Figure 5E. As shown in Figure 4C, the average height and roughness of the cell before treatment were 217.6 ± 25.8 and 129.2 ± 51.4 nm, respectively. In contrast, the average height and roughness of the treated cell were 559.8 ± 74.9 and 223.5 ± 14.1 nm, respectively (Figure 4D). As indicated by the red line, the corresponding cross and long sections of the cell surface before and after treatment were clearly different. Moreover, the C_14_-KYR-treated cell revealed more grooves and shrinkable cell surface than the untreated cell, as shown in profile 1 from Figure 4D,F, respectively. The blue and red arrows in Figure 4F indicate a major groove on the cell wall of the C_14_-KYR-treated cell, suggesting that this C_14_-KYR could severely damage the cell.

Figure 5 shows representative AFM deflection images of an *E. coli* cell incubated in the absence and presence of C_14_-KYR. When *E. coli* HB101 was exposed to C_14_-KYR for 15 min, the intercellular fluid was clearly visible around the apical of the cell, as indicated by the white arrow of Figure 5B,C. This result provides visual evidence of a disruption of the outer membrane of bacteria. Moreover, Figure 5D,E show that the C_14_-KYR induced disruption of the cytoplasmic inner membrane, whereas the outer membrane of the cell retained its rod shape, as observed in the untreated cell. Interestingly, the phase image (Figure 5D) shows the collapse of the inner cell membrane, while the outer membrane separated from the inner membrane. This result indicates that the C_14_-KYR induced the collapse of the inner membrane, which resulted in the bacteria losing control of the intracellular material. This observation may provide additional evidence for self-promoted uptake of the LiP toward the inner membrane, which possibly induced an incomplete loss of bacterial structure, leading to cell death. Figure 5E,F demonstrate the next stage of morphological change arising from a longer incubation time. When the *E. coli* HB101 cell was exposed to the C_14_-KYR peptide for 30 min, we observed a dramatic shrinkage and deep grooves on the surface presented in the inset of Figure 5E,F. These findings suggest that the creep deformation experiment can be an additional approach to provide visual and quantitative information to discern the differences between the mechanistic actions of different antimicrobial compounds. In this case, the AFM imaging showed that the C_14_-KYR caused severe permeabilization of the bacterial inner membrane.

C_14_-KYR first bound to the outer bacterial membrane that could permeabilize into the outer membrane. It further induced the inner bacterial membrane and completely depolarized the membrane afterward. Their action indicated that C_14_-KYR severely affected the viability of bacteria, resulting in death within 10 min [5]. In this study, visual evidence of the antibacterial mechanism especially revealed dramatic membrane deformation on the cell surface, inducing roughness on the surface by elongated, wrinkled, and severely ruptured cell morphology. Therefore, the AFM imaging evidence correlated with the mechanism of the lipopeptide’s membrane-permeabilization ability.

### 3.3. Rupture Force Measurements

**Figure 6:**
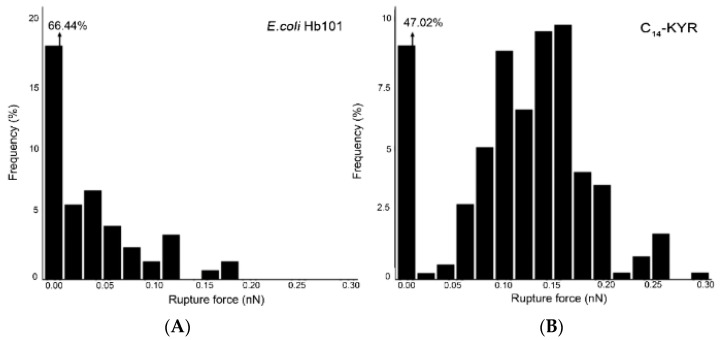
Histograms of frequency distribution of rupture force between the AFM tip and the *E. coli* HB101 in the absence (**A**) and presence of C_14_-KYR (**B**). For the untreated cell, the ruptured force ranged from ~0.02 to 0.18 nN. For the C_14_-KYR-treated cell, the rupture force ranged from ~0.02 to 0.3 nN. Each histogram corresponds to approximately 400 of single force curves.

Quantitative rupture force measurement with AFM is a promising way to characterize how the lipopeptide affects the mechanical properties of the bacterial surface. The AFM probe as nanoindentation depth plots was used and analyzed according to theoretical models for providing quantitative information on the rupture force of the sample [20]. These models have been applied toward bacteria, *E. coli* HB101. It was suggested that the elasticity of cells can change significantly depending on the lipopeptide in which the cells are exposed. Varying the absence and presence of C_14_-KYR at which the steric forces of the molecular substance on the bacterial membrane were measured, we then analyzed the produced force curves in Matlab (MathWorks, Natick, MA, USA) by using a procedure we previously described [14,29,30]. AFM-based force spectroscopy was applied to measure the rupture forces of the cell surface [38]. Interaction occurs between the biomolecule substances on the cell membrane and the AFM tip [39]. When the tip is brought into contact with the bacterial cell, repulsive interaction generally arises between the tip and sample, which are due to steric repulsion caused by the molecular substance on the bacterial membrane. Contact is achieved between the bacteria and the tip, and some of the bacterial molecule will remain adsorbed to the AFM tip because of intermolecular attraction [20]. Then, as the AFM tip is retracted from the surface, a force is required to separate the tip and the sample, and its magnitude is used to infer the force of bacteria to the lipopeptide. Herein, with the absence and presence of C_14_-KYR at MIC for 30 min (Figure 6), the sharp tip (about 10 nm in radius) was used to gently touch the bacterial surface in order to measure the rupture force. In the absence of C_14_-KYR, the observed rupture events ranged from ~0.02 to 0.18 nN with an average rupture force of 0.04 nN, corresponding to a 6.4% event frequency, Figure 6A. In the presence of C_14_-KYR at MIC (Figure 6B), the event frequency of the undetected rupture force (0 nN) was reduced ~1.4-fold from 66.4% to 47.0% compared to that of the untreated cell. The rupture force was observed in the range of ~0.02–0.3 nN. The average binding force was at 0.16 nN with the adhesion-rupture event frequency of 9.6%. Multiple binding events (3.7%) were also detected at forces greater than 0.2 nN. Basically, the rupture forces were associated with individual bond dissociation events. In these experiments, force plateaus were explained by the formation of lipid tethers that were pulled out of the lipid bilayers, with rupture occurring when the lipid reservoir was exhausted. The lipid tethers were estimated by dividing the breakage distance by the AFM retraction velocity. The existence of the high force region indicates that, to initiate the tether extraction process of the *E. coli* cell (untreated) case, substantial effort is needed. In contrast, due to C_14_ KYR bound to the outer bacterial membrane with electrostatic bonding between the positively charged KYR and the negatively charged bacterial membrane, we were able to permeate the inner membrane. This mechanism inducing a change in the bacterial morphology following treatment indicates membrane imbalances with the internal contents leaking out, accompanied by cell swelling, causing possible water uptake through cell lysis, which provides measurement of the lower rupture force.

In this study, intense damage of the *E. coli* HB101 cell surface was visualized after treatment with the C_14_-KYR lipopeptide. *E. coli* belongs to the Gram-negative bacteria consisting of a lipopolysaccharide outer membrane, thin peptidoglycan layer (~2 nm), and cytoplasmic membrane [40,41]. The lipopolysaccharide of the outer membrane of *E. coli* acts as a selective permeability barrier. As a result, when treated with 20 μM of KYR peptide, the peptide was unable to reach the peptidoglycan layer. Consequently, the *E. coli* HB101 remained in their rod-shaped structure, although some surface area was disrupted. In the other case, the concentration of 20 μM of C_14_-KYR lipopeptide could reach the peptidoglycan layer and into the inner membrane. The bacterial inner membrane shrank and became separated from the cell wall, resulting in the leakage of intracellular materials. The corresponding SR-FTIR results revealed structural changes in the membrane component after LiP attack. The most differences were especially found in the protein and peptide regions. This occurrence indicates that C_14_-KYR destabilizes the cell membrane by altering the structural conformation of membrane components resulting in membrane disruption and leakage. To further provide supporting evidence for the mechanistic action, the elasticity of the membrane surface was determined. We found that the static roughness of the *E. coli* surface after treatment with the C_14_-KYR became greater than that of the untreated cell. Additionally, the adhesion force between the biomolecule substance on the cell membrane and the AFM tip was determined from the rupture force analysis [29,38]. The highest frequency of the rupture force in the untreated *E. coli* cell was 0.04 nN, but with the treatment of C_14_-KYR, the rupture force increased to 0.16 nN. This finding indicated that the C_14_-KYR disrupted the bacterial membrane and induced changes in the elasticity of the cell surface, resulting in an alteration of their rod-shaped morphology and rigidity, which eventually led to cell death.

## 4. Conclusions

In our previous study, we highlighted and followed the progression of events that occur during the membrane disintegration process over time. We previously found that LiP, C_14_-KYR, exhibits higher antibacterial properties, particularly membrane-disrupting activity, than the parent peptide, KYR [5]. Here, we presented visual evidence for the antibacterial mechanism of the C_14_-KYR against *E. coli* HB101 and for the morphological changes in the cell surface using AFM imaging and IR spectroscopy. Measurements of the cell roughness and force spectroscopy were performed before and after exposure to the C_14_-KYR lipopeptide at different time courses. AFM creep deformation experiments of C_14_-KYR induced dramatic changes on the cell surface, including shrinkage of the cell surface, separation of the cell wall and cytoplasm, and leakage of intracellular materials. In contrast, cell treated with the KYR revealed a slightly damaged surface, giving a similar pattern to the untreated cell. Further evidence from SR-FTIR revealed structural changes in the membrane component after LiP attack. The most differences were especially found in the protein and peptide regions when exposed by 5x C_14_-KYR. This occurrence indicates that C_14_-KYR destabilizes the cell membrane by altering the structural conformation of membrane components, resulting in membrane disruption and leakage. This evidence provides detailed information on the morphological changes of the cell at the nanoscale, which corresponds to biochemical changes in the bacterial membrane.

## Figures and Tables

**Table 1:** Summary of properties of *E. coli* HB101 cell before and after peptide treatment.

Treatment Time	Sample	Size (µm)	Appearance Description of Cell Surface	Measurement (nm)		
				Mean of Height, *h*	The Average Roughness, Ra ^1^	RMS Roughness ^2^
0 min	Untreated (*n* = 251)	L: 3.4 ± 0.3 W: 1.43 ± 0.4	Smooth	310.4 ± 25.2	32.68 ± 4.7	43.03 ± 5.9
15 min	KYR (*n* = 176)	L: 2.8 ± 0.7 W: 1.28 ± 0.3	Smooth	471.0 ± 48.4	47.25 ± 9.7	63.3 ± 7.9
	C_14_-KYR (*n* = 213)	L: 2.4 ± 0.4 W: 1.34 ± 0.3	Rough	543.5 ± 32.2	179.67 ± 29.1	209.33 ± 33.8
30 min	KYR (*n* = 225)	L: 3.1 ± 0.3 W: 1.07 ± 0.1	slightly deformed surface	485.1 ± 35.3	68.03 ± 8.4	87.3 ± 14.3
	C_14_-KYR (*n* = 228)	L: 2.8 ± 0.5 W: 1.18 ± 0.12	Porous/Cell shrinkage	512.3 ± 14.2	176.44 ± 18.2	205.7 ± 20.5

L stands for length; W stands for width; n stands for number of *E. coli* HB101 cells. The measurement data were acquired from the analysis of cells from ≥8 independent cultures. ^1^ The average roughness (Ra) and ^2^ root-mean-square (RMS) roughness were measured in a scan size of 100 nm × 100 nm at the center of a single cell from each height image.

## Data Availability

The data presented in this study are available on the request from the corresponding author.

## Supplementary Materials

The following are available online at https://www.mdpi.com/article/10.3390/membranes11070495/s1, Figure S1: AFM image of a bare glass in 5 μm × 5 μm. Glass slides were cleaned by sonication for 15 min in 2% RBS 35 concentrate (Life technologies, NY, USA), and sequential washing was performed with 70% ethanol and ultrapure water (18.2 MΩcm resistivity and <10 ppb total organic carbon). Silicon nitride cantilevers (DNP-S, Veeco Instrument Inc., Santa Barbara, CA, USA) with a spring constant of ~0.12 N/m and a resonant frequency of 5 kHz were used in AFM imaging. The scanning speed was set to 0.5 Hz, Figure S2: The morphologies of *E. coli* HB101 without peptide treatments were measured for 0, 30, and 60 min. When the cell was not exposed to the peptides, its surface was relatively smooth without the appearance of ruptures or bulges. Total scanning area for each image: 5 × 5 μm, spring constant: ~0.09–0.12 N/m, resonance frequency: 24 kHz, scanning speed: 0.7 Hz.
