# Supplementary material for: AFM Study of Nanoscale Membrane Perturbation Induced by Antimicrobial Lipopeptide C14 KYR
Source: Membranes (Basel). 2021 Jun 30;11(7):495. doi: 10.3390/membranes11070495 (PMC8307486; doi:10.3390/membranes11070495)
Supplement: Supplementary file 1 [file membranes-11-00495-s001.zip › membranes-1255436-supplementary.pdf]

Supplementary data

AFM study of nanoscale membrane perturbation induced by antimicrobial lipopeptide C_{14}KYR

Sawinee Nasompag [1], Pawinee Siritongsuk [2], Saengrawee Thammawithan [2], Oranee Srichaiyapol [2], Panchika Prangkio [3], Terri A. Camesano [4], Chomdao Sinthuvanich[1,5] and Rina Patramanon [2,*]

[1] Interdisciplinary Graduate Program in Genetic Engineering, Graduate School, Kasetsart University, Bangkok 10900, Thailand; sawinee.nas@gmail.com
[2] Department of Biochemistry, Faculty of Science, Khon Kaen University, Khon Kaen 40002, Thailand; pawinee.siri@kkumail.com (P.S.); th_saengrawee@kkumail.com (S.T.); oranee_sr@kkumail.com (O.S.)
[3] Department of Chemistry, Faculty of Science, Chiang Mai University, Chiang Mai 50200, Thailand; panchika.p@cmu.ac.th
[4] Department of Chemical engineering, Worcester Polytechnic Institute, Massachusetts 01609, USA; terric@wpi.edu
[5] Department of Biochemistry, Faculty of Science, Kasetsart University, Bangkok 10900, Thailand; fscicds@nontri.ku.ac.th
* Correspondence: narin@kku.ac.th

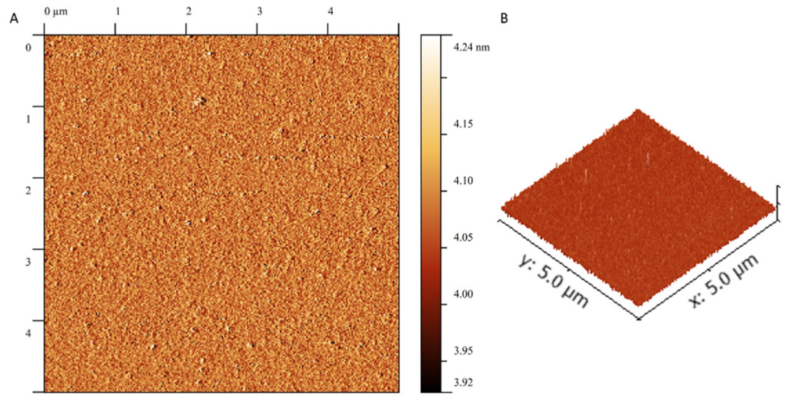

Figure S1. AFM image of a bare glass in 5 µm × 5 µm. Glass slides were cleaned by sonication for 15 min in 2% RBS 35 concentrate (Life technologies, NY, USA) and sequential washing was done with 70% ethanol and ultrapure water (18.2MΩcm resistivity and <10 ppb total organic carbon). Silicon nitride cantilevers (DNP-S, Veeco Instrument Inc., Santa Barbara, CA) with a spring constant of ~ 0.12 N/m and a resonant frequency of 5 kHz were used in AFM imaging. A scanning speed was set to 0.5 Hz.

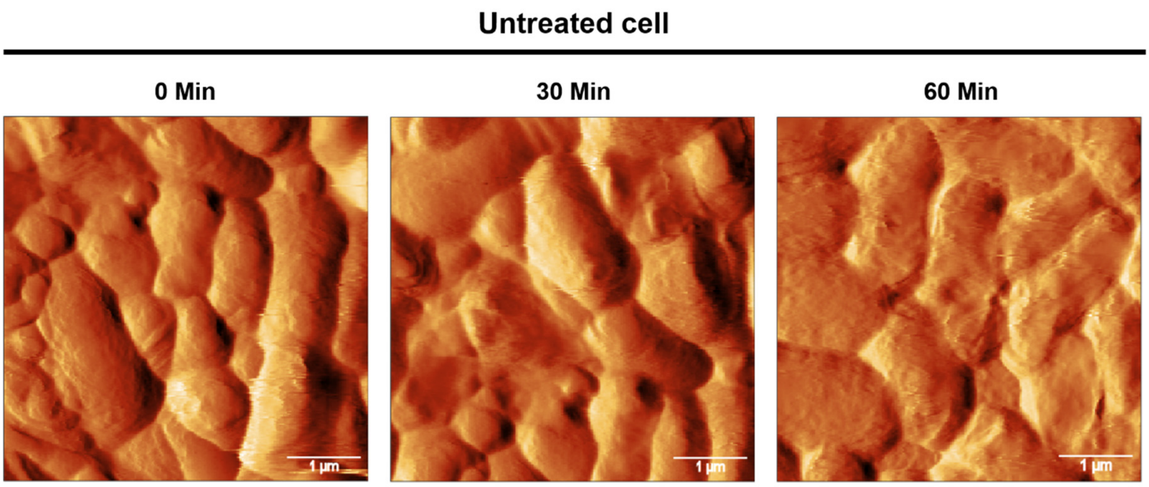

Figure S2. Morphology of *E. coli* HB101 without peptide treatments were measured for 0, 30 and 60 min. When the cell was not exposed to the peptides, its surface was relatively smooth without appearance of ruptures or bulges. Total scanning area for each image: 5 × 5 µm, spring constant ~0.12-0.14 N/m, resonance frequency 24 kHz and scanning speed was set to 0.7 Hz.
